# Loneliness and Its Association with Depression, Aspiration Risk, and Conversation in Japanese Older Adults

**DOI:** 10.3390/healthcare14020190

**Published:** 2026-01-12

**Authors:** Naoki Maki, Hitomi Matsuda, Sachie Eto, Akihiro Araki, Toshifumi Takao, Thomas Mayers

**Affiliations:** 1 Faculty of Rehabilitation, R Professional University of Rehabilitation, 2-12-31 Kawaguchi, Tsuchiura 300-0032, Ibaraki, Japan; 2 Department of Human Care Science, Graduate School of Comprehensive Human Sciences, University of Tsukuba, 1-1-1 Tennodai, Tsukuba 305-8575, Ibaraki, Japan; matsuda.hitomi.fn@u.tsukuba.ac.jp; 3 Department of Nursing, Faculty of Health Science Technology, Bunkyo Gakuin University, 1-19-1 Mukogaoka, Bunkyo 113-8668, Tokyo, Japan; s-eto@bgu.ac.jp; 4 Health and Nursing Administration, Oita University of Nursing and Health Sciences, 2944-9 Megusuno, Oita 870-1201, Oita, Japan; araki@oita-nhs.ac.jp; 5 Department of Physical Therapy, Faculty of Health Science, Tsukuba, International University, 6-8-33 Manabe, Tsuchiura 300-0051, Ibaraki, Japan; t-takao@tius.ac.jp; 6 Institute of Medicine, University of Tsukuba, 1-1-1 Tennodai, Tsukuba 305-8575, Ibaraki, Japan

**Keywords:** oral swallowing function, volunteering, older adults, frailty, depression, quality of life, loneliness, social isolation, social connection

## Abstract

**Background/Objectives:** Loneliness is a critical public health concern associated with adverse mental and physical health outcomes in later life. However, few large-scale studies have examined loneliness in relation to depression, aspiration risk, frailty, and social participation among Japanese older adults. This study examined associations between loneliness and psychosocial and health-related factors among older adults. **Methods:** This cross-sectional study involved a secondary analysis of data obtained from online surveys conducted in 2018 and 2021 among 1000 community-dwelling Japanese adults (≥65 years). Loneliness was assessed using the UCLA Loneliness Scale Version 3 and dichotomized at the median to define a high-loneliness group. Depressive symptoms, aspiration risk, frailty, conversation frequency, and volunteering participation were assessed using validated scales. Multivariable logistic regression was used to identify factors associated with loneliness and interaction terms were examined to assess effect modification. **Results:** High loneliness was observed in 52.2% of participants. Greater loneliness was significantly associated with depressive symptoms (GDS ≥ 5; OR = 4.69, 95% CI: 2.84–7.76), higher dysphagia risk (DRACE score; OR = 1.08, 95% CI: 1.00–1.16), and lower daily conversation frequency (OR = 0.76, 95% CI: 0.67–0.86); however, volunteering (OR = 0.475, 95% CI: 0.23–0.87) was a protective factor. **Conclusions:** Loneliness among Japanese older adults is closely linked to depressive symptoms and aspiration risk, while frequent conversations and volunteer participation appear to be protective. Community-based interventions promoting social engagement and oral health may mitigate loneliness and its health consequences and improve quality of life for older adults. Given the cross-sectional design, the observed associations should not be interpreted as causal.

## 1. Introduction

Loneliness among older adults is an urgent public health issue that threatens both mental and physical health [1,2]. In rapidly aging societies such as Japan, weakening of social connections is becoming increasingly evident, and this erosion of social ties heightens the risk of adverse health outcomes [3,4]. Moreover, prior research has shown that loneliness triggers chronic stress responses and adversely influences immune and cardiovascular systems, while social isolation and loneliness together are associated with mortality risk comparable to that of obesity and smoking [1,5,6]. Beyond depression, mounting evidence indicates that diminished social interaction is associated with impaired oral and swallowing function [7,8,9]. One Japanese longitudinal study involving 427 community-dwelling older adults found that multiple dimensions of oral hypofunction, including tooth loss, reduced occlusal force, impaired mastication, swallowing difficulties, and dry mouth, were significant predictors of worsening social withdrawal, with individuals exhibiting oral frailty at baseline being nearly twice as likely to become socially withdrawn over a two-year period [7]. Such declines in swallowing function can, in turn, contribute to malnutrition, frailty, aspiration pneumonia, impaired sleep quality, and general declines in quality of life (QOL), which are critical health risks for the well-being of older adults [10,11].

Conversely, activating social relationships can promote health, well-being, and QOL [12,13]. Daily conversations and interpersonal interactions provide cognitive stimulation and may prevent feelings of isolation and depression and even improve sleep quality [12,13], which accounts for the success of initiatives such as social prescribing that aim to improve well-being and QOL through increased social engagement and interactions [14,15,16]. Participation in volunteer activities or community programs not only strengthens social ties but also supports well-being and oral function by encouraging opportunities for speaking and eating together [16,17,18]. Volunteering has consistently been shown to offer numerous benefits, particularly for older adults [19,20,21,22,23,24,25]. The advantages of volunteering extend beyond emotional satisfaction; it plays a vital role in maintaining physical and mental health, including lower depressive symptoms, fewer functional limitations, and reduced mortality [16,19,25]. A large-scale longitudinal study in Japan also showed that voluntary work was protective against increasing levels of depression in older adults [23]. Likewise, our earlier findings among Japanese older adults indicated that individuals who volunteered experienced lower degrees of depression and loneliness than their non-volunteering peers [24]. Therefore, it seems reasonable to hypothesize that increased loneliness would contribute to poorer mental and physical health outcomes (higher depressive symptoms and possible aspiration risk) among older adults, while increased social interactions, frequent conversation, and volunteer activities would serve as protective social factors.

To address this topic, the present study utilized data from large-scale online surveys conducted in 2018 and 2021, encompassing 1000 community-dwelling older adults across Japan. This study aimed to clarify the associations between loneliness and key mental, physical, and social factors with a view to improving the long-term health, QOL, and well-being of older adults.

## 2. Materials and Methods

### 2.1. Participants

Data for the present study were derived from two previously conducted online surveys carried out in January 2018 and June 2021 [11,24]. Both surveys used identical procedures, instruments, and inclusion criteria, and were administered via the same national online survey platform. As the two datasets had been analyzed separately and published previously [11,24], this study represents a secondary re-analysis combining both cohorts to enable a more granular examination of factors associated with loneliness among community-dwelling older adults in Japan. Furthermore, pooling data from both phases provided a larger sample, thereby enhancing statistical power and the generalizability of findings. Because data collection protocols and psychometric measures were consistent across phases, combining the datasets was considered methodologically appropriate. The 2018 and 2021 surveys were conducted as independent, anonymous, cross-sectional surveys. Individual identifiers were not available; therefore, it was not possible to determine whether the same individuals participated in both surveys.

Participants were recruited through the online survey platform, Rakuten Insight (Rakuten Insight Global, Inc., Tokyo, Japan), an academic internet survey platform with over 2.2 million registered users across a wide demographic of ages and regions of Japan. This platform provided efficient access to a diverse study population while ensuring data quality, security, and compliance, and allowed for the collection of data that closely reflects Japan’s general population.

The inclusion criteria for participants were (1) age 65 years or older, (2) living independently in the community, and (3) ability to complete the questionnaire without assistance. Individuals with a physician-diagnosed dementia were excluded. A priori power analysis using G*Power (version 3.1.9.7, Düsseldorf, Germany) indicated that a minimum of 462 participants was required to achieve 95% power with an alpha level of 0.05 and a small-medium effect size [26].

### 2.2. Questionnaire

Demographic characteristics and lifestyle factors were assessed, including (1) sex and age, (2) height, weight, and body mass index (BMI), (3) living arrangements (living alone, with a partner, with a child/grandchild, or other), (4) current diseases under treatment, (5) daily activities (housework, volunteering), and (6) enjoyment (yes/no) and frequency of daily conversations. Participant age was recorded as self-reported chronological age at the time of each survey. Because the two surveys were independent cross-sectional samples, and individual participants could not be linked across survey waves, age was treated as age at survey and was not adjusted to a common reference year in the pooled dataset.

Participants’ physical and mental health status was comprehensively evaluated using the validated Japanese versions of the following five scales. The Japanese version of the Pittsburgh Sleep Quality Index (PSQI-J) was used to assess sleep disorders over the past month (score range 0–21, higher scores indicate worse sleep quality) [27,28]. The reliability of the Japanese version has been confirmed as high, indicated by a Cronbach’s alpha of 0.77 [28,29]. The Dysphagia Risk Assessment for Community-Dwelling Elderly (DRACE) instrument, a scale developed in Japan, was used to evaluate swallowing function and aspiration risk over the past year (12 items, total score 0–24; scores ≥ 5 indicate high dysphagia risk) [30]. The DRACE has shown good internal consistency, reflected by a Cronbach’s alpha of 0.88 [30]. The Japanese version of Geriatric Depression Scale-15 (GDS-15-J) was used to screen for depressive symptoms (score range 0–15; scores ≥ 5 indicate depression tendency) [31,32]. The Japanese version has shown strong internal consistency, with a Cronbach’s alpha of 0.83 [32]. The Japanese version of the University of California, Los Angeles Loneliness Scale Version 3 (UCLA-LS3-J) was used to measure loneliness (20 items, total score 20–80; higher scores indicate greater loneliness) [33,34]. The UCLA-LS3-J has demonstrated high reliability (α = 0.92) [34]. In this study, the median UCLA-LS3-J score was used as a cut-off to classify the participants into two groups: High-Loneliness Group and Low-Loneliness Group. The Kihon Checklist (KCL), developed by the Japanese Ministry of Health, Labour and Welfare, was used to assess frailty risk across seven domains including daily activities, nutrition, and swallowing (25 items, score range 0–25; higher scores indicate greater frailty) [35]. A previous study has established the KCL’s effectiveness in identifying frailty among older adults [35]. Table S1 summarizes the measurement instruments, scoring ranges, and interpretative criteria for the key study variables used in the analyses.

### 2.3. Statistical Analysis

Participant characteristics were summarized as means ± standard deviations or numbers (*n*) and percentages (%). Group differences between the High- and Low-Loneliness Groups were evaluated using the chi-squared test for categorical variables and the Mann–Whitney U test for continuous variables. The Mann–Whitney U test was selected for group comparisons because several continuous variables exhibited skewed distributions and/or ordinal scale characteristics. Binary logistic regression analyses were performed to identify factors associated with high loneliness. Model I was adjusted for age and sex only. Model II was a theory-driven multivariable model including depressive symptoms, dysphagia risk, conversation frequency, volunteer participation, sleep disorder, and survey year. Survey year was dummy-coded (2018 = 0, 2021 = 1).

Multicollinearity was assessed using variance inflation factors (VIFs), and no substantial multicollinearity was detected. Interaction terms between survey year and age, survey year and sex, and between volunteering and conversation frequency were examined to assess potential effect modification [36]. Odds ratios (OR) with 95% confidence intervals (CI) were calculated. A two-tailed *p* value of <0.05 was considered statistically significant. Statistical analyses were performed using SPSS version 29.0.1.0 (IBM Corporation, Armonk, NY, USA).

### 2.4. Ethics

This study was conducted in accordance with the principles of the Declaration of Helsinki. Informed consent was obtained electronically from all participants prior to participation, as outlined by Rakuten Insight. This study was approved by the Ethics Committee of the Institute of Medicine, University of Tsukuba (Approval No. 1623-5, date 24 October 2022).

## 3. Results

A total of 1000 community-dwelling older adults participated in the survey, comprising 477 males (47.7%) and 523 females (52.3%). Based on the a priori power analysis, the final sample size of 1000 provided sufficient statistical power to detect meaningful differences. The mean age of the entire cohort was 72.8 ± 5.6 years, and the average BMI was 22.7 ± 3.3 kg/m^2^. Based on the UCLA-LS3-J scores, participants were divided into a High-Loneliness Group (*n* = 522) and a Low-Loneliness Group (*n* = 478) for analysis. Table 1 summarizes the demographic and clinical characteristics of the participants. There were no significant differences in living arrangements between the two groups; approximately two thirds of the cohort lived with a partner or family members, and the proportions of those living alone or in couple-only households did not differ significantly between groups. Similarly, BMI showed no significant group difference (*p* = 0.649).

Significant psychosocial differences emerged between the groups; participants in the High-Loneliness Group were significantly older (73.44 ± 5.13 vs. 72.26 ± 5.11 years, *p* < 0.001) and more often female (56.8% vs. 43.2%, *p* = 0.002). The High-Loneliness Group reported markedly lower frequency of daily conversations (3.77 ± 1.96 vs. 4.84 ± 1.63 times/day, *p* < 0.001) and a lower enjoyment in engaging in conversations (36.8% vs. 63.1%, *p* < 0.001). Participation in volunteer activities was significantly less common among those in the High-Loneliness Group (32.4% vs. 67.6%, *p* < 0.001).

Regarding psychosomatic status, the High-Loneliness Group had substantially higher GDS-15-J scores (5.08 ± 3.00 vs. 2.88 ± 2.13, *p* < 0.001) and a greater prevalence of depressive symptoms (GDS ≥ 5: 77.6% vs. 22.4%, *p* < 0.001). The DRACE scores, reflecting pulmonary aspiration risk, were also higher in the High-Loneliness Group (2.77 ± 3.16 vs. 1.91 ± 2.40, *p* < 0.001), and a greater proportion had DRACE scores ≥4 (62.5% vs. 37.5%, *p* < 0.001) indicating higher risk of pulmonary aspiration. Sleep quality measured by the PSQI was slightly worse in the High-Loneliness Group (5.17 ± 2.76 vs. 4.52 ± 2.26, *p* = 0.017), although the prevalence of sleep disorder (PSQI ≥ 6) remained modest (16.5% overall). Frailty, as assessed with the KCL, was also more common in the High-Loneliness Group (mean score 7.65 ± 4.76 vs. 6.50 ± 4.33, *p* < 0.001).

The UCLA-LS3-J results clearly distinguished the two groups (49.41 ± 5.64 vs. 34.82 ± 5.79, *p* < 0.001), confirming the internal consistency of the group classification. Table 2 shows diseases under current treatment; hypertension (41.1%), diabetes mellitus (12.8%), and lower back pain (11.9%) were the most frequent conditions, but none differed significantly between the two groups. No significant interactions were observed between survey year and sex, survey year and age, or between volunteering and conversation frequency; therefore, stratified analyses were not performed.

The multivariable logistic regression (Table 3) identified several independent correlates of high loneliness. Greater depressive symptoms (GDS ≥ 5) were strongly associated with high loneliness (OR = 4.69, 95% CI: 2.84–7.76, *p* < 0.001). Higher DRACE scores were also independently related to high loneliness (OR = 1.08 per point, 95% CI: 1.00–1.16, *p* = 0.042). Conversely, more frequent daily conversations showed a protective effect (OR = 0.76 per additional conversation, 95% CI: 0.67–0.86, *p* < 0.001), as was participation in volunteer activities (OR = 0.45, 95% CI: 0.23–0.87, *p* = 0.017). Female sex was independently associated with lower odds of high loneliness (OR = 0.44, 95% CI: 0.29–0.66, *p* = 0.001), whereas survey year was a significant predictor, with higher odds of loneliness observed in 2018 compared with 2021. Age and sleep disorder were not significantly associated with high loneliness in the fully adjusted model. These findings indicate that, as suspected, depressive symptoms, aspiration risk, and limited conversational engagement are key factors associated with higher loneliness among Japanese older adults. In terms of effect size, depressive symptoms showed the strongest association with high loneliness, with an OR of 4.69, indicating a substantially higher likelihood of loneliness among participants with depressive symptoms. Higher aspiration risk was also associated with increased odds of loneliness, whereas more frequent conversation and participation in volunteer activities were associated with meaningfully lower odds of high loneliness.

**Figure 1:**
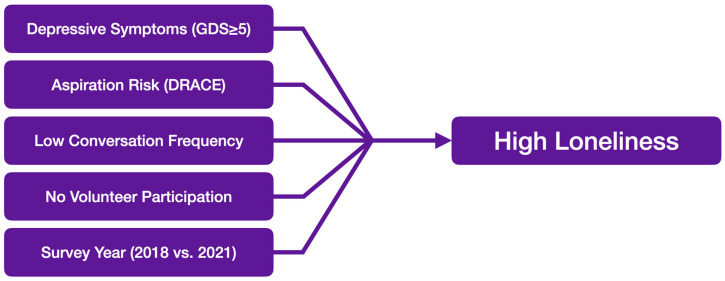
Logistic regression analysis. Notes: GDS, Geriatric Depression Scale; DRACE, Dysphagia Risk Assessment for Community-Dwelling Elderly.

Figure 1 provides a conceptual summary of factors independently associated with high loneliness identified in the final multivariable model.

## 4. Discussion

The findings of this study indicate that older adults with higher loneliness scores were at a clear disadvantage regarding both mental health and oral swallowing function outcomes. Participants in the High-Loneliness Group exhibited significantly higher depressive symptoms, poorer swallowing function, and lower conversation frequency, whereas frequent conversation and participation in volunteer activities were strongly associated with lower loneliness scores. Several variables that were significant in bivariate analyses lost significance after multivariable adjustment, suggesting confounding or shared variance with other psychosocial factors rather than independent effects.

A particularly important implication of this study is the close association between oral–swallowing function and social engagement. Declines in swallowing ability have been reported to be associated not only with an increased risk of malnutrition and aspiration pneumonia but also with reduced social participation, as older adults may avoid eating with others due to the fear of coughing or choking in public [9,37,38]. Such avoidance behaviors may be associated with fewer opportunities for shared meals and conversation, thereby exacerbating loneliness and further reducing oral activity. A recent study from Singapore found that older adults with chewing disabilities had a 48% higher risk of clinically significant depressive symptoms and loneliness accounted for a meaningful portion of this association, mediating approximately 17% of the total effect [39]. Conversely, interventions that maintain or enhance oral and swallowing function, such as tongue and swallowing exercises, individualized oral care, and regular dental checkups, may be associated with reducing anxiety about eating with others, increasing opportunities for dining out, and naturally stimulating conversation.

Participants with higher loneliness scores had significantly higher GDS-15-J scores and were more likely to meet criteria for depressive symptoms. This is consistent with previous research demonstrating that loneliness could increase the risk of depression, cognitive decline, and mortality [1,9,40,41,42,43,44,45,46]. From a mechanistic perspective, loneliness has been linked to stress-related biological pathways, including dysregulation of the hypothalamic–pituitary–adrenal axis, chronic inflammation, and impaired immune responses, which may in turn exacerbate depressive symptoms and physical frailty [47,48]. Interestingly, our data add to this growing body of research by showing that swallowing dysfunction is also an integral, albeit often overlooked, part of the pathway linking loneliness and mental health, suggesting that interventions aimed at oral and swallowing function [11,49,50,51,52] could simultaneously improve psychological well-being. Moreover, diminished desire or ability to converse may be further influenced by oral functional decline, such as swallowing problems, reduced articulatory capacity, oral discomfort, and dental problems, which can make speaking effortful or embarrassing, thereby reinforcing social withdrawal, loneliness, and depressive symptoms [13].

Volunteer activity emerged as another protective factor against loneliness. Consistent with earlier reports [13,19,24,53], participation in volunteering offers older adults a sense of purpose and social usefulness, enhances self-efficacy, and provides structured opportunities for regular interaction. These psychological and social benefits are likely to have an association with lower levels of loneliness and better mental health. Our previous study found that volunteers reported significantly lower depression and loneliness and greater social and hobby engagement than non-volunteers, supporting evidence that volunteering may serve as a low-cost strategy to enhance mental health and social participation in older adults [24]. Furthermore, volunteer settings often involve communal meals, refreshments, or informal gatherings where participants can enjoy speaking, eating, and drinking in a socially supportive environment. Such experiences may indirectly strengthen oral function by promoting regular use of oral musculature during conversation and eating. The finding that volunteer participation was independently associated with lower loneliness in our regression analysis indicates the potential relevance of community-based volunteer programs as a public health intervention. In the Japanese cultural context, community-based volunteering and neighborhood activities are an important means for maintaining social connectedness among older adults, particularly after retirement. Such activities often involve face-to-face interaction, shared meals, and regular conversation, which are culturally embedded practices that may help to mitigate loneliness and improve QOL in later life.

Volunteering is particularly motivating for older adults because it fulfills several core psychological and social needs that are closely tied to well-being, purpose, and healthy aging. A substantial body of research shows that volunteer activity supports eudaimonic well-being, providing a sense of meaning, productivity, and social value that becomes especially important in later life when traditional roles (e.g., employment or caregiving) may diminish [54,55]. Engaging in altruistic, prosocial behavior has been linked to enhanced self-efficacy, greater perceived control, and stronger feelings of social connectedness [19,56] of which eating is an important, spiritual and physical component. A review by Anderson et al. found evidence that volunteering may be a particularly powerful lifestyle activity for healthy aging, as evidence from observational and experimental studies consistently shows that formal volunteer work is associated with reduced depression, better self-rated health, fewer functional limitations, and lower mortality, likely through increased social, cognitive, and physical engagement that could ultimately help mitigate functional decline and possibly reduce dementia risk [19]. 

Volunteering also offers structured and predictable opportunities for interaction, which have the potential to reduce loneliness and promote a sense of belonging through regular contact with others who share similar goals or interests [53,57]. These motivational benefits, namely, purpose, altruism, usefulness, and companionship, are powerful drivers of continued engagement in volunteer activities. Moreover, longitudinal research indicates that volunteers often experience lower levels of depression, better cognitive trajectories, and even reduced mortality risk, suggesting that the meaningful contributions they make generate reinforcing cycles of psychological and physical resilience [21,58]. Furthermore, these findings align with broader research on social connectedness, including sociotype-based approaches that examine how the structure and quality of daily social interactions relate to loneliness and health outcomes in older adults. For example, Navarro et al. proposed that reduced conversational engagement and weakened social networks are key components of a disrupted sociotype, which is strongly associated with loneliness and poorer health in later life [59].

In our study, DRACE scores, indicative of dysphagia risk, showed a modest association with loneliness. While this finding should be interpreted cautiously given the proximity of the confidence interval to the null value, it suggests the usefulness of interventions aimed at maintaining oral and swallowing function together with social engagement as possible preventive measures against loneliness (and its associated physical and mental declines). Programs that include nutrition guidance, oral exercises [60,61,62], and community-based volunteer opportunities, e.g., Refs. [15,24,63] may simultaneously protect swallowing function, stimulate daily conversation, and alleviate loneliness [64,65,66]. Local governments could partner with dental associations and senior centers to create regular opportunities for group dining and oral exercise sessions, ensuring that older adults maintain both physical and social skills necessary for healthy aging.

Another important implication is the need for early detection and monitoring of loneliness. The UCLA Loneliness Scale used in this study provides a validated tool for identifying individuals at risk [33,34]. Incorporating such assessments into routine health checkups or dental visits could help clinicians identify older adults who may benefit from targeted interventions. Dentists and dental hygienists, who frequently observe changes in oral function, are in a unique position to screen for loneliness and provide referrals to community programs. For example, in a large-scale Japanese study, lonely older adults were significantly more likely to experience tooth loss, infrequent toothbrushing, and chewing difficulties, demonstrating how oral-health assessments can reveal underlying psychosocial vulnerabilities that may otherwise go unnoticed [67]. Indeed, collaborative care models in which dentists, physicians, speech-language pathologists, and mental health professionals share information and coordinate treatment could be particularly effective for older adults at high risk of both oral decline and psychological distress.

Another noteworthy observation that arose from our data was that frailty scores, as measured by the KCL, were higher in the High-Loneliness Group. Frailty encompasses physical, psychological, and social dimensions, and our findings suggest that loneliness may be both a cause and a consequence of frailty. This finding is in line with a large body of research that has pointed to this bidirectional association, e.g., Refs. [68,69,70,71]. Oral frailty, which refers to a decline in oral function that precedes physical frailty, has increasingly been recognized as an important public health concern in Japan [72]. For example, a Japanese longitudinal study by Kusunoki and colleagues, which used the Oral Frailty Five-Item Checklist (OF-5), demonstrated that oral frailty is closely linked to reduced muscle mass, slower walking speed, and poorer physical function, and that higher OF-5 scores predict subsequent progression of physical frailty, findings that strongly suggest the importance of early oral-frailty screening in preventing broader functional decline among older adults [72]. By linking oral frailty to loneliness, our study provides further justification for policies that prioritize oral health as a key component of healthy aging [73].

An important finding was the independent association between survey year and loneliness. Although overall mean loneliness scores were lower in 2021, the odds of belonging to the High-Loneliness Group were higher. This apparent discrepancy may indicate a difference in loneliness experiences among older adults during the COVID-19 period, with reductions observed for some individuals alongside a concentration of high loneliness among more vulnerable subgroups. Interaction analyses indicated that this temporal pattern did not differ by age or sex within the study sample, suggesting that the pandemic-related increase in loneliness was broadly observed across demographic groups.

Policy implications from this study are considerable. Municipalities and national governments should consider further incorporating oral health promotion (including dental care and oral exercises) and social participation (such as social prescribing interventions) into community care systems for older adults [73].

### Limitations

This study has several limitations that should be considered when interpreting the findings. First, the cross-sectional design prevents determination of causal relationships among loneliness, depression, oral–swallowing function, and social participation. Second, all data were collected through self-administered online questionnaires, which may introduce selection bias. Older adults with lower digital literacy, more severe physical disabilities, or advanced cognitive impairment were likely underrepresented. This potential selection bias should be considered when interpreting the findings, particularly with respect to alternative forms of social contact during the COVID-19 period. The COVID-19 pandemic was period characterized by reduced opportunities for face-to-face interactions and substantial changes in social behavior and daily routines. These pandemic-related contextual factors may have influenced loneliness, social participation, and conversational frequency, and therefore should be considered when interpreting differences between survey years. Third, the assessments of oral and swallowing function were based on validated self-report instruments rather than direct clinical examinations. While tools such as the DRACE have demonstrated reliability, objective clinical measures (such as tongue pressure, oral diadochokinesis, or fiberoptic endoscopic evaluation) would provide more precise evaluation of swallowing ability. Future research could incorporate both self-reported and objective assessments to strengthen the validity of findings. Fourth, while we analyzed a wide range of health and lifestyle factors, unmeasured confounding variables, such as socioeconomic status, education level, nutritional intake, or access to dental care, may have influenced the observed associations. These factors should be included in future models to better understand the pathways linking oral function, social engagement, and loneliness. Also, potential endogeneity cannot be ruled out, as loneliness and depressive symptoms likely influence each other. Fifth, loneliness was dichotomized using a median cut-off to define relatively high and low loneliness groups. Because no validated clinical cut-off has been established for the UCLA Loneliness Scale Version 3 in community-dwelling populations, this approach was adopted to facilitate interpretability and comparison of relative risk groups, consistent with prior epidemiological research. However, this dichotomization may have resulted in some loss of information and should be interpreted as reflecting relative, rather than clinical, levels of loneliness [45]. Sixth, although the surveys were designed as independent cross-sectional studies, the possibility of duplicate participation across survey waves cannot be completely excluded. Finally, the generalizability of our findings to other cultural contexts may be limited. Social dining customs, volunteer opportunities, and oral/dental health practices vary internationally. Systematic reviews and comparative studies in diverse cultural settings are necessary to confirm whether the potential pattern of oral health, social participation, and reduced loneliness observed in this Japanese cohort applies to other aging societies.

## 5. Conclusions

This large-scale, cross-sectional study identified significant associations among frailty, loneliness, depressive symptoms, oral–swallowing function, and social participation in community-dwelling older adults. High loneliness scores were found to be associated with greater depressive symptoms, reduced swallowing function, and fewer opportunities for conversation, whereas frequent conversation and participation in volunteer activities had possible protective effects. Given the cross-sectional design, the observed associations should not be interpreted as causal relationships; however, our findings imply that oral health is not only a determinant of physical well-being but also a key factor in sustaining social engagement and psychological resilience. Maintaining oral and swallowing function could reduce fear of coughing or choking during meals, encourage dining with others, and stimulate meaningful conversation. Such interactions may reflect a positive pattern of association: enhanced oral function leads to more shared meals and conversations, which in turn help preserve oral function, and reduce loneliness and depressive symptoms. Interventions that combine oral health promotion, nutrition guidance, and opportunities for volunteer activity may therefore constitute relevant components of strategies to prevent depression and improve QOL in older adults. From a public health perspective, integrating oral health programs with community-based social participation initiatives should be considered in policies for promoting healthy aging. Municipalities, healthcare providers, and dental professionals can work together to implement early screening, oral exercise programs, and community dining events to support both physical and mental health. By breaking the cycle of oral decline, social withdrawal, and loneliness, such interventions could potentially help older adults maintain independence, social connectedness, QOL, and overall well-being throughout later life.

## Figures and Tables

**Table 1:** Participant demographic characteristics.

		Total (*n* = 1000) *n* (%)	High-Loneliness Group (*n* = 522) *n* (%)	Low-Loneliness Group (*n* = 478) *n* (%)	*p*
Age (Years)		72.76 ± 5.57	72.26 ± 5.11	73.44 ± 5.13	<0.001 **
Gender	Male	477 (47.7%)	225 (47.2%)	252 (52.8%)	0.002 **
	Female	523 (52.3%)	297 (56.8%)	226 (43.2%)	
BMI		22.69 ± 3.28	22.71 ± 3.39	22.70 ± 3.03	0.649
Family:					
Living alone	Yes	342 (34.2%)	204 (39.0%)	138 (28.9%)	0.081
	No	658 (65.8%)	318 (61.0%)	340 (71.1%)	
With a child/grandchild	Yes	154 (15.4%)	80 (15.3%)	74 (15.4%)	0.706
	No	846 (84.6%)	442 (84.7%)	404 (84.6%)	
With a partner	Yes	430 (43.0%)	184 (35.2%)	246 (51.4%)	0.676
	No	570 (57.0%)	338 (64.8%)	232 (48.6%)	
Others	Yes	74 (7.4%)	38 (7.3%)	36 (7.5%)	0.428
	No	926 (92.6%)	484 (92.7%)	442 (92.5%)	
Daily activities:					
Housework	Yes	693 (69.3%)	363 (52.4%)	330 (47.6%)	0.863
	No	307 (30.7%)	159 (51.8%)	148 (48.2%)	
Volunteer	Yes	111 (11.1%)	36 (32.4%)	75(67.6%)	<0.001 **
	No	889 (88.9%)	486 (54.7%)	403 (45.3%)	
Enjoys conversation	Yes	584 (58.4%)	215 (36.8%)	369 (63.1%)	<0.001 **
	No	408 (40.8%)	299 (73.2%)	109 (26.7%)	
Frequency of conversations/day		4.28 ± 1.88	3.77 ± 1.96	4.84 ± 1.63	1
GDS-15-J score		4.03 ± 2.84	5.08 ± 3.00	2.88 ± 2.13	<0.001 **
Depression (GDS ≥ 5)	Yes	255 (25.5%)	198 (77.6%)	57 (22.4%)	<0.001 **
	No	745 (74.5%)	324 (43.5%)	421 (56.5%)	
PSQI-J		4.78 ± 2.50	5.17 ± 2.76	4.52 ± 2.26	0.017 *
Sleep disorder (PSQI ≥ 6)	Yes	165 (16.5%)	83 (50.3%)	82 (49.7%)	0.008 **
	No	335 (33.5%)	127 (37.9%)	208 (62.1%)	
DRACE score		2.36 ± 2.85	2.77 ± 3.16	1.91 ± 2.40	<0.001 **
Pulmonary aspiration risk, DRACE ≥ 4	Yes	264 (26.4%)	165 (62.5%)	99 (37.5%)	<0.001 **
	No	736 (73.6%)	357 (48.5%)	379 (51.5%)	
UCLA-LS3-J		42.40 ± 9.31	49.41 ± 5.64	34.82 ± 5.79	<0.001 **
KCL		7.10 ± 4.59	7.65 ± 4.76	6.50 ± 4.33	<0.001 **
Frailty KCL ≥ 5	Robust	348 (34.8%)	155 (29.7%)	193 (40.4%)	<0.001 **
	Prefrail/frail	652 (65.2%)	367 (70.3%)	285 (59.6%)	

* *p* < 0.05; ** *p* < 0.01, Mean ± SD, χ^2^ test, Mann–Whitney U test; Abbreviations: PSQI-J, Pittsburgh Sleep Quality Index Japanese version; DRACE, Dysphagia Risk Assessment for Community-Dwelling Elderly instrument; GDS-15-J, Geriatric Depression Scale-15 Japanese version; UCLA LS3-J, University of California, Los Angeles Loneliness Scale 3 Japanese version; KCL, Kihon Checklist.

**Table 2:** Diseases under treatment among the study cohort.

	Total (*n* = 1000) *n* (%)	High-Loneliness Group (*n* = 522) *n* (%)	Low-Loneliness Group (*n* = 478) *n* (%)	*p*
Hypertension	329 (41.1)	172 (21.5)	157 (19.6)	0.265
Heart disease	67 (8.4)	32 (4.0)	35 (4.4)	0.238
Arteriosclerosis	19 (2.4)	8 (1.0)	11 (1.4)	0.267
Cerebrovascular disease	14 (1.8)	9 (1.1)	5 (0.6)	0.464
Pneumonia	10 (1.3)	4 (0.5)	6 (0.8)	0.350
Asthma	25 (3.1)	11 (1.4)	14 (1.8)	0.278
Chronic Obstructive Pulmonary Disease	6 (0.8)	1 (0.1)	5 (0.6)	0.061
Diabetes mellitus	102 (12.8)	53 (6.6)	49 (6.1)	0.563

**Table 3:** Logistic regression analysis.

Variables	Model I ^†^ OR (95% CI)	*p*	Model II ^‡^ OR (95% CI)	*p*
Age (per year)	0.98 (0.95–1.02)	0.271	0.98 (0.95–1.02)	0.271
Sex (female)	0.44 (0.29–0.66)	<0.001	0.44 (0.29–0.66)	<0.001
Depression (GDS ≥ 5)	—	—	4.69 (2.84–7.76)	<0.001
DRACE score (per point)	—	—	1.08 (1.00–1.16)	0.042
Conversation frequency (per day)	—	—	0.76 (0.67–0.86)	<0.001
Volunteer (yes)	—	—	0.45 (0.23–0.87)	0.017
Sleep disorder (yes)	—	—	1.01 (0.65–1.58)	0.382
Survey year (2018 vs. 2021)	—	—	1.74 (1.32–2.29)	<0.001

Abbreviations: OR, odds ratio; CI, confidence interval; GDS, Geriatric Depression Scale; DRACE, Dysphagia Risk Assessment for Community-Dwelling Elderly. ^†^ Model I adjusted for age and sex. ^‡^ Model II mutually adjusted for all variables listed. Survey year was dummy-coded (2018 = 0, 2021 = 1). High loneliness was defined using the median cut-off of the loneliness scale.

## Data Availability

The original contributions presented in the study are included in the article/Supplementary Materials, further inquiries can be directed to the corresponding authors.

## Supplementary Materials

The following supporting information can be downloaded at https://www.mdpi.com/article/10.3390/healthcare14020190/s1, Table S1: Definition and measurement of study variables.
