# Supplementary material for: Loneliness and Its Association with Depression, Aspiration Risk, and Conversation in Japanese Older Adults
Source: Healthcare (Basel). 2026 Jan 12;14(2):190. doi: 10.3390/healthcare14020190 (PMC12841345; doi:10.3390/healthcare14020190)
Supplement: Supplementary file 1 [file healthcare-14-00190-s001.zip › healthcare-4053920-supplementary.pdf]

**Table S1**. Definition and measurement of study variables

| Variable | Instrument / Scale | Range / Cut-off | Interpretation |
| --- | --- | --- | --- |
| Loneliness | UCLA LS 3-J | 20–80 (median split) | Higher scores indicate greater loneliness |
| Depression | GDS-15-J | ≥5 | Depressive symptoms present |
| Aspiration risk | DRACE | 0–12 | Higher scores indicate greater risk |
| Conversation frequency | Self-reported | Categorical | Higher frequency indicates more frequent conversation |
| Volunteer participation | Self-reported | Yes / No | Participation in volunteer activities |
| Frailty | KCL | 0–25 | Higher scores indicate greater frailty |
| Survey year | Survey wave | 2018 / 2021 | Year of data collection |

Abbreviations: UCLA LS3-J, University of California, Los Angeles Loneliness Scale 3 Japanese version; GDS-15-J, Geriatric Depression Scale-15 Japanese version; DRACE, Dysphagia Risk Assessment for Community-Dwelling Elderly instrument; KCL, Kihon Checklist.
